# Enzymatic Bioremodelers: From Molecular Mechanisms to Clinical Applications in Aesthetic and Regenerative Medicine

**DOI:** 10.1111/jocd.71180

**Published:** 2026-10-06

**Authors:** Antony de Paula Barbosa

**Affiliations:** ^1^ Department of Research and Development Antony Barbosa Institute (ABI) Belo Horizonte Minas Gerais Brazil; ^2^ Pontifícia Universidade Católica de Minas Gerais (PUC Minas) Belo Horizonte Minas Gerais Brazil

**Keywords:** adipose tissue, aesthetic medicine, collagenase, enzymatic bioremodelers, extracellular matrix, fibrosis, hyaluronidase, lipase, recombinant enzymes, regenerative medicine

## Abstract

**Background:**

The steady evolution of aesthetic and regenerative medicine has driven a paradigm shift from purely corrective interventions toward biologically oriented therapies focused on tissue remodeling, extracellular matrix modulation, and regenerative optimization. Recent advances in biotechnology and recombinant protein engineering have enabled the development of highly purified enzymes that selectively interact with structural and functional components of the tissue microenvironment. Despite their growing clinical relevance, therapeutic enzymes are still often discussed in isolation, limiting a broader understanding of their shared biological mechanisms and therapeutic potential.

**Materials and Methods:**

A narrative review was conducted, integrating evidence from molecular biology, extracellular matrix research, fibrosis, adipose tissue physiology, protein therapeutics, regenerative medicine, and aesthetic medicine. In addition to foundational biological studies, recent clinical and translational investigations involving recombinant hyaluronidases, collagenases, lipases, keratinases, and matrix‐targeting lyases were analyzed. Evidence from scar management, post‐liposuction fibrosis, localized adiposity, cellulite, facial reshaping, foreign body granulomas, periocular edema associated with hyaluronic acid fillers, and biostimulator‐related complications was critically reviewed.

**Results and Discussion:**

The available evidence demonstrates that therapeutic enzymes share common biological characteristics, including substrate‐specific molecular recognition, catalytic activity, and selective modulation of tissue morphology. Clinical studies have reported improvements in fibrosis, scar quality, cellulite, localized fat deposits, facial contouring, tissue laxity, granulomatous reactions, and filler‐related complications. Histological analyses additionally support their capacity to reorganize extracellular matrix structures, reduce fibrosis, improve collagen organization, and restore tissue homeostasis. Unlike conventional therapies that rely primarily on inflammation, cytotoxicity, or mechanical disruption, enzyme‐based interventions promote targeted biochemical remodeling through precise substrate interactions.

**Conclusion:**

The collective evidence supports the establishment of Enzymatic Bioremodelers as a novel therapeutic category comprising natural or recombinant enzymes that promote selective tissue remodeling using targeted biochemical modulation of the tissue microenvironment. This framework integrates molecular mechanisms with emerging clinical applications, providing a scientifically grounded platform for future developments aesthetic medicine, regenerative medicine, and precision therapeutics.

## 1 Introduction

Over the past two decades, aesthetic medicine has undergone a major transformation. Initially focused on correcting visible signs of aging through surgical procedures, volume replacement, and superficial cosmetic interventions, the field has evolved toward biologically driven strategies to improve tissue quality, restore structural strength, and modulate the physiological processes underlying aging and tissue degeneration. This transition has been largely supported by advances in regenerative medicine, molecular biology, biomaterials science, and a growing understanding of the extracellular matrix (ECM) as a dynamic regulator of tissue homeostasis rather than a passive structural scaffold [1, 2, 3, 4].

The extracellular matrix plays a central role in maintaining tissue framework and function. Beyond its structural role, the ECM actively participates in cell signaling, mechanotransduction, angiogenesis, immune regulation, and tissue repair. Its composition, which includes collagens, elastin fibers, glycosaminoglycans, proteoglycans, and matricellular proteins, continuously changes throughout life in response to physiological and pathological stimuli [1, 2]. Aging, fibrosis, chronic inflammation, adipose tissue dysfunction, and wound healing abnormalities are all associated with alterations in extracellular matrix organization and composition, making the ECM a major therapeutic target in both regenerative and aesthetic medicine [2, 3, 4].

The search for therapeutic methods that selectively modify tissue morphology has led to the development of numerous interventions, including biostimulators, energy‐based devices, mechanical remodeling techniques, cell‐based therapies, and injectable agents. Although these modalities have demonstrated clinical utility, most achieve their effects through indirect mechanisms such as controlled inflammation, mechanical disruption, thermal injury, or stimulation of fibroblast activity [5, 6, 7, 8]. In contrast, enzyme‐based therapies offer a fundamentally different biological approach. Rather than inducing secondary tissue remodeling, enzymes act directly on specific molecular substrates, producing highly targeted biochemical modifications within the tissue microenvironment.

Enzymes are biological catalysts that accelerate chemical reactions through substrate‐specific molecular recognition and catalytic activity. Their therapeutic use has expanded considerably in recent decades due to advances in recombinant DNA technology, protein engineering, and large‐scale biopharmaceutical production [9, 10]. Recombinant proteins are currently among the fastest‐growing therapeutic classes in modern medicine, with applications ranging from oncology and endocrinology to hematology and rare genetic disorders [9]. Within dermatology and aesthetic medicine, however, the potential of therapeutic enzymes remains underexplored and often misunderstood.

Historically, the term “enzymes” has been used inconsistently in aesthetic practice. In many countries, especially throughout Latin America, the term became associated with injectable formulations marketed for localized fat reduction and body contouring, regardless of whether true enzymatic molecules were present. Many of these formulations consisted of pharmacological compounds, detergents, methylxanthines, phosphatidylcholine, deoxycholic acid, or various mesotherapy cocktails with no genuine enzymatic activity. As a result, the term gradually lost its biochemical meaning and became disconnected from its scientific definition. This semantic confusion has resulted in a fragmented understanding of enzyme‐based therapies and has limited the development of a coherent biological framework for their clinical application.

Recent advances in recombinant biotechnology have renewed interest in therapeutic enzymes as highly specific tools for tissue remodeling. Unlike classic injectable formulations, recombinant enzymes are engineered proteins produced by controlled biotechnological processes, allowing high purity, reproducibility, substrate specificity, and predictable biological behavior [10]. These characteristics have enabled the advancement of novel therapeutic approaches based on selective modulation of extracellular matrix components, fibrotic tissue, adipose compartments, and other structural elements of the tissue microenvironment.

Among the enzymes currently investigated or applied in aesthetic and regenerative medicine, hyaluronidases, collagenases, lipases, keratinases, and matrix‐targeting lyases have appeared as particularly relevant. Hyaluronidases selectively degrade hyaluronic acid, facilitating extracellular matrix reorganization and serving as crucial tools for managing complications associated with hyaluronic acid fillers [11, 12, 13, 14]. Collagenases can degrade pathological collagen accumulations and have demonstrated success in fibrotic disorders such as Dupuytren's contracture and Peyronie's disease, as well as potential in scar remodeling and aesthetic applications [15, 16, 17]. Lipases catalyze triglyceride hydrolysis and have recently been investigated as alternatives to adipocytolytic agents for localized fat reduction and body contouring [18, 19, 20, 21]. Keratinases and matrix‐targeting lyases further expand the scope of enzymatic intervention by acting on keratinized structures, proteoglycans, and glycosaminoglycans that are involved in tissue organization and regeneration [22, 23, 24, 25].

Importantly, growing clinical evidence suggests that the therapeutic role of recombinant enzymes goes beyond isolated molecular targets. Recent clinical studies have demonstrated positive results in the treatment of hypertrophic, atrophic, and keloid scars, showing improvements on the Vancouver and POSAS scales, as well as in vascularity, pigmentation, pliability, and scar relief following treatment with recombinant enzymatic combinations associated with hyaluronic acid. Additional studies have documented significant clinical and histological improvements in patients with post‐liposuction fibrosis, including reductions in inflammatory infiltrates, improved collagen organization, restoration of elastic fiber integrity, and enhanced skin quality following the application of combinations of recombinant collagenase, lipase, and lyase.

An important practical aspect observed across the available clinical studies is the relatively consistent interval adopted between treatment sessions. Most published protocols employ treatment intervals ranging from approximately 21 to 30 days. Although this schedule has not been formally established through pharmacokinetic investigations, it appears to be supported primarily by biological and clinical considerations rather than by enzyme half‐life alone. Following enzymatic modulation of extracellular matrix components, tissues undergo sequential phases of inflammatory resolution, extracellular matrix reorganization, fibroblast adaptation, collagen turnover, and biomechanical stabilization before additional intervention is performed. Allowing approximately 3 weeks between sessions may therefore optimize tissue remodeling while minimizing cumulative inflammatory responses and improving treatment tolerability. Nevertheless, comparative clinical studies evaluating different treatment intervals remain unavailable, and future investigations should establish evidence‐based treatment schedules for each clinical indication [26, 27, 28, 29, 30, 31, 32].

Similarly, prospective clinical investigations have shown the efficacy of recombinant enzymatic mixtures in managing moderate‐to‐severe submental fat, with significant reductions in fat pad thickness confirmed by ultrasonography and high patient satisfaction rates. Other reports have documented positive results in cellulite treatment, foreign body granulomas, periocular edema following hyaluronic acid filler injections, and even complications associated with polycaprolactone‐based collagen stimulators, showing the broad translational potential of enzyme‐based therapies throughout diverse clinical scenarios.

Despite their diverse indications, these enzymes share a common biological principle. Their therapeutic effects are mediated through selective catalytic interactions with specific molecular substrates, resulting in targeted remodeling of structural and functional tissue components. This shared mechanism distinguishes them from conventional therapies based on inflammation, cytotoxicity, adipocytolysis, or mechanical disruption. Rather than acting through generalized tissue injury, these enzymes operate through biochemical precision, making them inherently compatible with the principles of precision medicine and regenerative therapeutics.

The emergence of this body of evidence suggests the need for a wider conceptual framework that integrates these therapies into a common biological and mechanistic classification. We propose that these agents should be understood as members of a distinct therapeutic category termed EBs, defined as natural or recombinant catalytic biological agents capable of promoting selective tissue remodeling using targeted biochemical modulation of structural and functional components of the tissue microenvironment.

Therefore, the objective of this review is to examine the molecular foundations, biological mechanisms, and clinical evidence supporting enzyme‐mediated tissue remodeling, and to propose a unified conceptual framework for EBs. Through integrating data from extracellular matrix biology, fibrosis research, adipose tissue physiology, protein therapeutics, and emerging clinical applications, this review intends to establish a scientific basis for understanding the role of therapeutic enzymes in aesthetic and regenerative medicine and to provide a basis for future research, clinical innovation, and translational development.

## 2 Materials and Methods

This study was conducted as a narrative review, using a translational and conceptual approach to explore the biological foundations, molecular mechanisms, and clinical applications of therapeutic enzymes in aesthetic and regenerative medicine. Given the emerging nature of the proposed concept and the heterogeneity of the available evidence, a narrative methodology was selected to integrate data from molecular biology, extracellular matrix research, fibrosis studies, adipose tissue physiology, regenerative medicine, protein therapeutics, and clinical aesthetic practice [33, 34]. The methodological organization and reporting of the narrative review were additionally guided by principles proposed for the quality assessment of narrative review articles, including the SANRA framework [35].

A comprehensive literature review was performed using publications indexed in PubMed/MEDLINE, Scopus, Web of Science, and Embase. Additional evidence was identified through manual screening of reference lists from relevant publications and through the analysis of recent clinical studies, observational investigations, case series, and translational reports involving recombinant enzyme therapies. The search strategy focused on therapeutic enzymes and tissue remodeling and included terms associated with hyaluronidase, collagenase, lipase, keratinase, lyase, extracellular matrix, fibrosis, scar remodeling, adipose tissue, regenerative medicine, recombinant proteins, and precision therapeutics.

The selection process prioritized peer‐reviewed scientific articles, clinical trials, prospective and retrospective studies, translational investigations, consensus documents, and landmark publications that advanced understanding of enzyme‐mediated tissue remodeling. Classical studies addressing extracellular matrix biology, wound healing, fibrosis, adipose tissue metabolism, and protein engineering were included to provide the biological rationale for the proposed framework. In addition, recent publications that specifically evaluate recombinant enzyme therapies for scar treatment, post‐liposuction fibrosis, cellulite, localized adiposity, facial reshaping, foreign body granulomas, periocular edema following hyaluronic acid filler injections, wound healing, and biostimulator‐related complications were incorporated to support the clinical translation of the concept.

The reviewed evidence was analyzed qualitatively and interpreted in terms of its contribution to the understanding of catalytic tissue modulation. Particular attention was given to substrate specificity, mechanisms of enzymatic action, interactions with extracellular matrix components, effects on fibrosis and tissue repair, modulation of adipose tissue composition, regenerative potential, safety profile, and clinical relevance. Rather than focusing exclusively on treatment outcomes, the review sought to identify common biological principles shared across enzyme classes and to establish mechanistic links between molecular activity and clinical tissue remodeling.

The available evidence was subsequently organized into interconnected thematic domains, including extracellular matrix remodeling, fibrotic tissue modulation, adipometabolic remodeling, regenerative tissue repair, protein therapeutics, and clinical aesthetic applications. Via comparative analysis of these domains, recurrent mechanistic patterns were identified, particularly substrate‐specific molecular recognition, catalytic precision, and selective modification of structural and functional tissue components.

Based on the combination of experimental, translational, and clinical evidence, a conceptual framework was developed to define a novel therapeutic category termed EBs. Within this system, EBs are understood as natural or recombinant catalytic biological agents that promote selective tissue remodeling using targeted biochemical modulation of the tissue microenvironment. The framework was constructed to bridge molecular biology, regenerative medicine, extracellular matrix science, and clinical aesthetics, providing a biologically grounded classification system for current and future enzyme‐based therapeutic methods.

Because the main objective of this work was conceptual and hypothesis‐generating, no quantitative synthesis or meta‐analysis was performed. Instead, the evidence was critically evaluated and integrated to support the biological plausibility, mechanistic coherence, and translational relevance of EBs as an emerging therapeutic platform in aesthetic and regenerative medicine.

## 3 Results and Discussion

### 3.1 Biological Foundations of Enzymatic Bioremodelers

The progressive evolution of aesthetic and regenerative medicine has been accompanied by a growing understanding that tissue aging, fibrosis, structural deterioration, and adipose tissue dysfunction are fundamentally biological processes rather than merely anatomical alterations. This paradigm shift has encouraged the development of therapeutic methods that interact directly with the molecular and structural components underlying tissue framework and function. Within this context, the concept of EBs presents a fresh framework that integrates enzyme‐based interventions based on the common biological mechanism rather than their individual clinical indications (Table 1).

**TABLE 1:** Proposed biological domains of action of enzymatic bioremodelers (EBs).

Biological domain	Main target	Representative enzyme(s)	Clinical outcomes
Extracellular matrix remodeling	Hyaluronic acid, glycosaminoglycans, proteoglycans	Hyaluronidase, Lyase	Tissue reorganization, edema reduction, ECM normalization
Fibrotic tissue remodeling	Type I and III collagen	Collagenase	Scar remodeling, fibrosis reduction, improved tissue elasticity
Adipometabolic remodeling	Triglycerides within adipocytes	Lipase	Localized fat reduction, body contouring, submental remodeling
regenerative remodeling	ECM signaling pathways and cellular interactions	Combined enzymatic therapies	Improved skin quality, elasticity, and tissue homeostasis
complication remodeling	Fillers, granulomas, fibrosis, biostimulator nodules	Hyaluronidase, Collagenase, Lipase	Resolution of adverse events and restoration of normal tissue architecture

*Note:* Proposed Definition: Enzymatic Bioremodelers are natural or recombinant catalytic biological agents that promote selective tissue remodeling through targeted biochemical modulation of the structural and functional components of the tissue microenvironment. *Source:* Elaborated by the author.

The extracellular matrix (ECM) occupies a central role in tissue homeostasis and represents one of the primary targets of EBs. Traditionally regarded as a passive scaffold, the ECM is now recognized as a highly dynamic and biologically active structure that regulates cellular adhesion, migration, differentiation, mechanotransduction, angiogenesis, and immune responses [1, 2]. Its composition includes collagens, elastin fibers, glycosaminoglycans, proteoglycans, and multiple signaling molecules that continuously interact with resident cells to maintain tissue cohesion and performance.

During aging and pathological remodeling, considerable alterations occur in the extracellular matrix. These include collagen fragmentation, elastin degradation, abnormal accumulation of glycosaminoglycans, fibrosis, and alterations in tissue hydration and biomechanics [2, 3, 4]. Such alterations contribute directly to skin laxity, scar formation, cellulite, adipose tissue dysfunction, and loss of structural support. Consequently, therapeutic methods that selectively modify extracellular matrix components have become increasingly attractive in both regenerative and aesthetic medicine.

Unlike conventional aesthetic interventions, which commonly rely on inflammatory stimulation, thermal injury, mechanical disruption, or volumetric replacement, EBs act through direct catalytic interactions with specific molecular substrates. This characteristic provides a degree of biological accuracy that distinguishes them from most currently available tissue remodeling therapies. Through substrate recognition and enzymatic catalysis, EBs can selectively modify structural tissue components while limiting collateral tissue damage. The therapeutic use of proteolytic enzymes for selective tissue modification is not unprecedented, as enzymatic debridement has long demonstrated that catalytic agents can selectively remove devitalized tissue while facilitating wound‐bed preparation and subsequent tissue repair [36].

From a biochemical perspective, the defining characteristic of EBs is substrate specificity. Enzymes possess highly organized active sites that recognize specific molecular structures and catalyze precise biochemical reactions. This substrate specificity allows selective modulation of extracellular matrix molecules, fibrotic tissue components, adipose tissue triglycerides, keratinized proteins, and glycosaminoglycans. Consequently, the therapeutic effects of EBs are determined not by generalized tissue injury but by targeted molecular associations that ultimately result in tissue remodeling.

The development of recombinant protein technologies has additionally expanded the therapeutic potential of EBs. Recombinant enzymes are produced by controlled biotechnological processes that allow high purity, reproducibility, and predictable biological behavior. Advances in molecular engineering have enabled the production of enzymes with optimized catalytic activity, improved stability, reduced immunogenicity, and enhanced substrate specificity, characteristics that are indispensable for safe and effective clinical application [9, 10].

Importantly, the biological rationale of EBs goes beyond simple substrate degradation. Tissue remodeling is a highly integrated process involving architectural reorganization, cellular responses, mechanical adaptation, and restoration of tissue homeostasis. By modifying key components of the tissue microenvironment, enzymes can indirectly influence fibroblast activity, inflammatory signaling, angiogenesis, adipose tissue metabolism, and extracellular matrix turnover. Therefore, the therapeutic significance of EBs consists not only in their catalytic activity but also in their capacity to initiate broader biological remodeling processes.

The convergence of extracellular matrix biology, protein engineering, regenerative medicine, and clinical aesthetics provides a scientific basis for considering EBs as a distinct therapeutic category. Although individual enzymes differ substantially in substrate specificity and clinical indications, they share common mechanistic principles: catalytic precision, substrate selectivity, and the ability to induce targeted remodeling of structural and functional tissue components. This commonality supports their classification within a consolidated framework and establishes the biological foundation for the concept of Enzymatic Bioremodelers (Figure 1).

**FIGURE 1:**
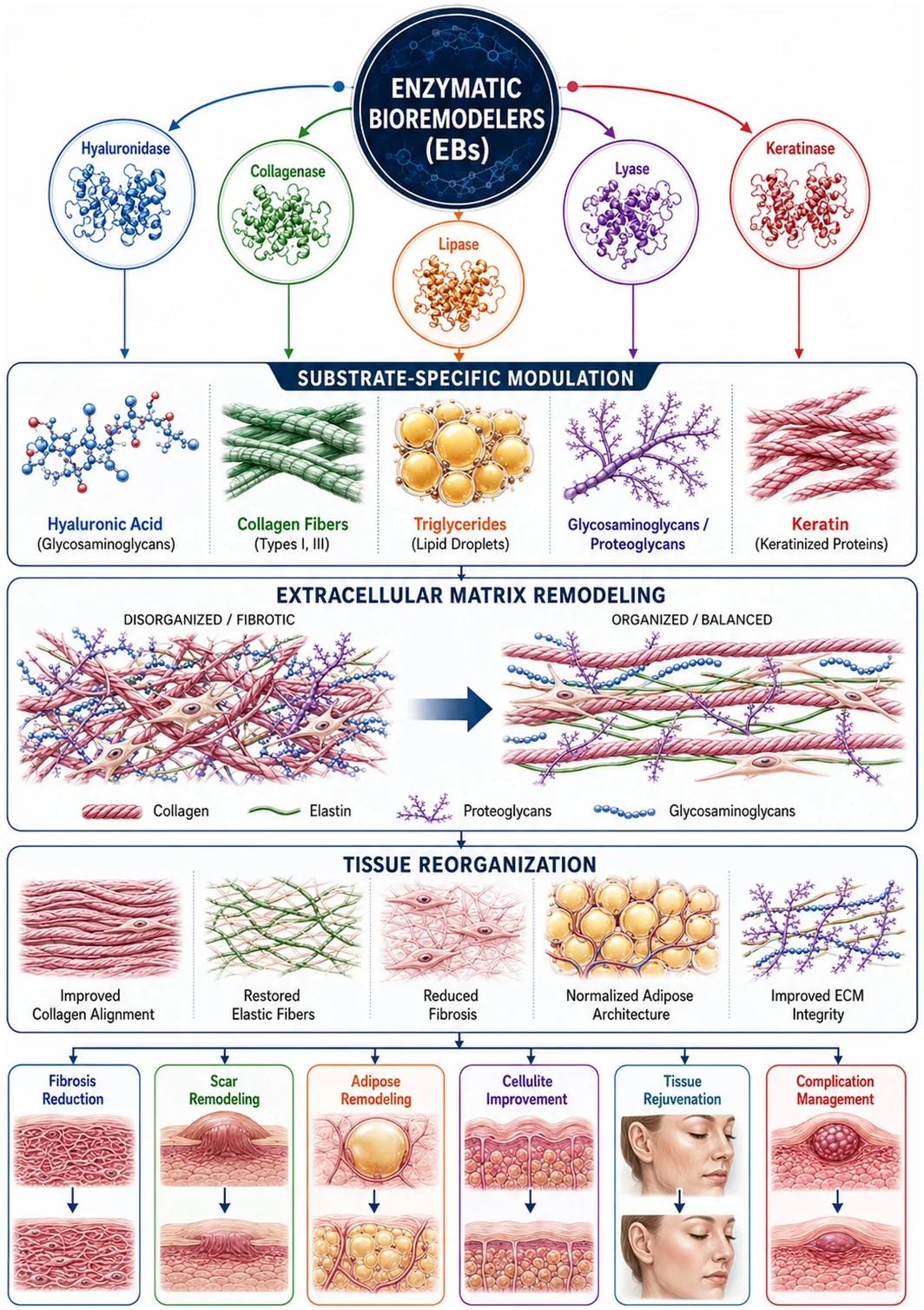
Proposed mechanistic model of Enzymatic Bioremodelers (EBs). Hyaluronidases, collagenases, lipases, lyases, and keratinases promote substrate‐specific modulation of extracellular matrix components, fibrotic tissue, adipose tissue, and keratinized structures [11, 12, 13, 14, 15, 16, 17, 18, 19, 20, 21, 22, 23, 24, 25]. Through selective catalytic activity, these enzymes induce extracellular matrix remodeling and tissue reorganization, resulting in clinically demonstrated outcomes such as fibrosis reduction, scar remodeling, adipose remodeling, cellulite improvement, tissue rejuvenation, and management of aesthetic complications [37, 38, 39, 40, 41, 42, 43]. Elaborated by the author with the assistance of artificial intelligence tools.

### 3.2 Hyaluronidase: The Prototype of Enzymatic Bioremodelers

Among all enzymes currently used in aesthetic medicine, hyaluronidase represents the most established and clinically validated example of an EB. Its therapeutic effects result from the selective degradation of hyaluronic acid, one of the principal glycosaminoglycans of the extracellular matrix [11, 12, 13, 14].

Hyaluronic acid plays a key role in tissue hydration, viscoelasticity, cellular communication, and extracellular matrix organization. Through its remarkable capacity to retain water, it contributes significantly to tissue volume, resilience, and mechanical function. Physiologically, hyaluronic acid undergoes continuous synthesis and degradation, maintaining a dynamic equilibrium that is essential for tissue homeostasis [11].

Hyaluronidases catalyze the hydrolysis of glycosidic bonds within hyaluronic acid chains, resulting in decreased molecular size, reduced matrix viscosity, increased tissue permeability, and enhanced diffusion of biological molecules. Although these biochemical effects have been recognized for decades, their importance in aesthetic medicine has become especially apparent with the widespread use of hyaluronic acid fillers [12].

Clinically, hyaluronidase is widely regarded as the gold standard for managing complications associated with hyaluronic acid fillers, including vascular compromise, nodules, overcorrection, persistent edema, and the Tyndall effect [11, 12, 13, 14]. However, its role stretches far beyond simple filler dissolution. By selectively modifying one of the major structural components of the extracellular matrix, hyaluronidase actively promotes tissue reorganization and restoration of physiological architecture.

Recent clinical evidence has expanded the possible applications of hyaluronidase beyond filler reversal. Castelanich et al. demonstrated that high‐dose hyaluronidase combined with collagenase effectively improved late‐onset periocular edema following hyaluronic acid filler injections, whilst avoiding post‐hyaluronidase syndrome, a recently recognized complication associated with repeated enzymatic degradation of endogenous extracellular matrix hyaluronic acid. Significant aesthetic improvement was observed in all treated patients, with no major complications reported. These data reinforce the concept that hyaluronidase functions as a true bioremodeling agent rather than merely a corrective enzyme. Through selective modulation of the extracellular matrix, it contributes to tissue normalization, restoration of hydraulic dynamics, and optimization of tissue quality. Consequently, hyaluronidase serves as the prototype for comprehending the broader concept of EBs.

### 3.3 High‐Molecular‐Weight Hyaluronic Acid as a Biomimetic Extracellular Matrix Hydrogel

Although high‐molecular‐weight hyaluronic acid (HMW‐HA) has traditionally been described as a pharmaceutical excipient or carrier in recombinant enzymatic formulations, this definition incompletely represents its biological significance. Current evidence demonstrates that native HMW‐HA is an active structural and signaling component of the extracellular matrix (ECM), playing a role in tissue hydration, viscoelasticity, mechanotransduction, interstitial fluid homeostasis, and immune regulation through interactions with CD44 and other hyaladherins [44, 45, 46, 47, 48]. Therefore, the incorporation of HMW‐HA into recombinant enzymatic formulations should be interpreted not only from a pharmaceutical perspective but also within the wider context of extracellular matrix biology.

This concept is notably relevant when HMW‐HA is combined with recombinant lyases. From a biochemical standpoint, hyaluronan lyases catalyze the cleavage of hyaluronic acid through a β‐elimination mechanism, generating unsaturated oligosaccharides rather than hydrolytic products [11, 44]. Consequently, recombinant lyases are capable of degrading hyaluronic acid under appropriate biochemical conditions, and HMW‐HA should not be regarded as intrinsically resistant to enzymatic activity. However, the therapeutic rationale for combining recombinant lyase with HMW‐HA does not rely on the assumption that the hydrogel is protected from degradation.

Following intratissue administration, recombinant lyase is immediately exposed to a highly complex extracellular matrix containing abundant endogenous hyaluronan, chondroitin sulfate, proteoglycans, and other glycosaminoglycan‐rich structures that constitute its physiological substrates [11, 44]. At present, no experimental studies have demonstrated preferential or complete degradation of the exogenously administered HMW‐HA contained within recombinant enzymatic formulations under clinical conditions. Therefore, although partial enzymatic cleavage of exogenous HMW‐HA cannot be excluded, the currently available evidence does not support considering the hydrogel merely as a sacrificial substrate or assuming that its biological contribution is immediately abolished after injection.

Instead, HMW‐HA provides immediate biological and physicochemical functions during enzyme delivery. Native HMW‐HA preserves extracellular matrix hydration, regulates interstitial fluid flow, contributes to tissue viscoelasticity, and maintains extracellular matrix homeostasis through CD44‐mediated signaling pathways [45, 46, 47]. In contrast, fragmentation of hyaluronan into low‐molecular‐weight species is associated with activation of Toll‐like receptors (TLR2 and TLR4), NF‐κB signaling, and amplification of inflammatory responses [45, 46, 47]. This molecular weight‐dependent functional dichotomy is now recognized as a fundamental principle of hyaluronan biology.

Accordingly, HMW‐HA should be regarded as a biologically active extracellular matrix‐mimetic hydrogel that establishes a hydrated and homeostatic microenvironment during localized enzymatic remodeling. Simultaneously, recombinant lyase promotes selective cleavage of endogenous hyaluronan and susceptible glycosaminoglycan‐rich extracellular matrix components, thereby increasing matrix permeability and assisting controlled tissue remodeling [11, 44]. Rather than representing biologically opposing mechanisms, these components perform complementary functions through integrating selective extracellular matrix remodeling with preservation of tissue framework and physiological homeostasis.

Importantly, the rationale for combining recombinant lyase with HMW‐HA should not be interpreted as an attempt to prevent enzymatic degradation of the hydrogel itself. Rather, extracellular matrix remodeling and extracellular matrix homeostasis are concurrent and complementary biological processes during tissue repair. Within this context, HMW‐HA functions as a biologically active extracellular matrix‐mimetic biomaterial, while recombinant lyase selectively remodels endogenous extracellular matrix substrates, together creating a controlled microenvironment favorable for tissue remodeling.

Although dedicated mechanistic studies investigating enzyme–hydrogel interactions in recombinant formulations are still necessary, the current understanding of extracellular matrix biology provides a biologically plausible rationale supporting the incorporation of HMW‐HA into recombinant enzymatic formulations beyond its traditional pharmaceutical role [44, 45, 46, 47].

To our knowledge, no published study has specifically investigated the kinetics of recombinant PB72K in the presence of HMW‐HA within the commercial formulation. Consequently, the proposed rationale is based on established principles of hyaluronan biology and extracellular matrix remodeling rather than on formulation‐specific kinetic studies.

Although recombinant lyases and hyaluronidases catalyze the breakdown of hyaluronic acid, their catalytic mechanisms and biological outcomes differ fundamentally from classical mammalian hydrolases [11, 45, 46, 47, 48, 49, 50, 51]. Classical hyaluronidases hydrolyze β‐1,4‐glycosidic bonds, whereas hyaluronan lyases employ a β‐elimination mechanism that cleaves the hexosaminidic C‐O bond, generating non‐inflammatory Δ^4,5^‐unsaturated oligosaccharides [11, 45, 46, 47, 48, 49, 50, 51].

Within co‐formulated enzymatic systems, High‐Molecular‐Weight Hyaluronic Acid (HMW‐HA, > 1.0 MDa) plays a dual biomimetic and physiological role [48, 49]. Functionally, native HMW‐HA restores interstitial fluid homeostasis, maintains tissue viscoelasticity, and forms a hydrophilic scaffolding [48, 49]. Biologically, HMW‐HA binds CD44 receptors to promote tissue survival and downregulate pro‐inflammatory signaling pathways, in stark contrast to Low‐Molecular‐Weight HA (LMW‐HA) fragments that activate TLR2/TLR4 and NF‐κB inflammatory cascades [46, 49, 50].

Although enzymatic degradation of exogenously administered HMW‐HA may occur progressively, the complex endogenous extracellular matrix—abundant in endogenous hyaluronan, chondroitin sulfate, and proteoglycans—competes for the enzyme's active site [49, 51]. This competitive substrate availability may allow HMW‐HA to exert its structural and CD44‐mediated homeostatic effects during the initial phase of tissue remodeling before complete enzymatic resolution occurs [48, 49, 50].

### 3.4 Collagenases and Fibrotic Tissue Remodeling

If hyaluronidase exemplifies extracellular matrix modulation through glycosaminoglycan degradation, collagenases represent the paradigm of fibrotic tissue remodeling. Collagen is the most abundant protein in the human body and serves as the primary structural element responsible for tissue strength and mechanical support. Under physiological conditions, collagen synthesis and degradation remain tightly regulated. However, pathological processes such as fibrosis, hypertrophic scarring, keloid formation, and chronic inflammation disturb this balance, resulting in excessive collagen accumulation and abnormal extracellular matrix organization [3, 4].

Collagenases are proteolytic enzymes capable of degrading fibrillar collagen through selective cleavage of the collagen triple helix. Their therapeutic relevance was initially established in conditions such as Dupuytren's contracture and Peyronie's disease, where pathological collagen accumulation results in significant functional impairment [15, 16, 17].

Within recombinant enzymatic formulations targeting fibrotic tissue, collagenase activity relies on a synergistic dual‐enzyme system comprising Collagenase G (Class I) and Collagenase H (Class II) derived from recombinant *Clostridium histolyticum* [52]. Although both zinc‐dependent metalloendopeptidases target the triplet Pro‐X‐Gly‐Pro sequence unique to collagen, they exert distinct yet interdependent catalytic processivities [52].

Collagenase G (Class I) features a dedicated Collagen‐Binding Domain (CBD) that recognizes and anchors to native, intact, triple‐helical collagen fibrils (Types I and III). Class I catalyzes internal endopeptidase cleavages within the tightly wound triple helix, effectively unlocking dense structural fibers into smaller intermediate fragments [52]. Conversely, Collagenase H (Class II) exhibits preferential affinity for terminal regions of collagen chains and newly exposed fragment ends, acting processively from the terminal regions to further degrade the fragments into smaller peptides [52].

The combination of Class I and Class II at an optimized stoichiometric mass ratio (approximately 1:3) overcomes the catalytic rate‐limiting step of collagen breakdown. Internal cleavage by Class I continually generates new terminal substrates for Class II, accelerating the total dissolution of pathological fibrosis while preventing the persistence of semi‐degraded, immunogenic collagen breakdown products [52].

The biological significance of collagenases goes beyond collagen degradation itself. Through disrupting pathological extracellular matrix networks, these enzymes modify tissue biomechanics, alter mechanotransduction pathways, facilitate fibroblast reorganization, and create conditions favorable for physiological remodeling. Therefore, collagenase‐mediated remodeling should be understood as a process of architectural reorganization rather than simple tissue degradation.

Recent evidence suggests that recombinant collagenases may play an important role in aesthetic and regenerative medicine. A multicenter study involving 42 patients with 44 fibrotic scars showed significant improvements in pigmentation, vascularity, pliability, and scar relief following treatment with combinations of recombinant collagenase, lipase, lyase, and hyaluronic acid. Improvements were observed using both the Vancouver Scar Scale and POSAS assessment, with favorable safety outcomes.

Similarly, clinical and histological analyses of post‐liposuction fibrosis showed significant tissue remodeling following the application of recombinant enzymatic therapy. Histological examination demonstrated reduced collagen basophilia, decreased inflammatory infiltrates, improved collagen organization, and restoration of elastic fiber integrity, findings that support a genuine remodeling effect rather than simple tissue degradation.

Collectively, these observations position collagenases as one of the most important components of the EB platform, particularly in conditions characterized by pathological fibrosis, scar formation, and extracellular matrix disorganization.

### 3.5 Lipases and the Concept of Adipometabolic Remodeling

Among the enzymes included within the proposed EB framework, lipases introduce one of the most innovative and potentially transformative concepts in aesthetic medicine. Whereas established approaches to localized adiposity primarily rely on adipocyte destruction or the physical removal of adipose tissue, lipases offer a fundamentally different biological strategy that selectively modulates lipid metabolism.

Traditionally, the treatment of localized fat deposits has focused on procedures such as liposuction, cryolipolysis, radiofrequency‐assisted lipolysis, high‐intensity focused ultrasound, and injectable adipocytolytic agents. Most of these approaches achieve volume reduction through direct adipocyte injury, membrane disruption, apoptosis, or mechanical extraction of adipose tissue [18, 19, 20, 21]. Injectable deoxycholic acid, for example, acts through detergent‐mediated disruption of adipocyte membranes, resulting in cell lysis, inflammation, and subsequent clearance of cellular debris [18]. Although clinically effective, this mechanism is intrinsically dependent on tissue injury and inflammatory responses.

Lipases operate through a markedly different biological pathway. These enzymes catalyze the hydrolysis of triglycerides into glycerol and free fatty acids, representing a central step in physiological lipid metabolism [15, 16, 19]. Rather than targeting cellular membranes, lipases act directly on stored lipids, potentially allowing modification of adipose tissue without immediate adipocyte destruction. This distinction forms the basis of what may be described as adipometabolic remodeling, a process in which tissue architecture is modified through metabolic regulation rather than cytolysis.

Modern adipose tissue biology supports this concept. Adipose tissue is now recognized as a complex endocrine and immunometabolic organ that actively participates in systemic homeostasis by producing adipokines, cytokines, angiogenic factors, and extracellular matrix components [6, 22, 23]. Consequently, therapeutic strategies directed at adipose tissue should be evaluated not only by volume reduction but also by their capacity to modulate tissue biology and restore physiological function.

Recent clinical evidence provides preliminary support for this hypothesis. In a prospective cohort study involving patients with moderate‐to‐severe submental fat, treatment with a recombinant enzymatic mixture containing lipase, collagenase, and hyaluronidase resulted in significant reductions in submental fat thickness as measured by ultrasonography. More than 90% of patients achieved clinically meaningful improvement on both clinician‐ and patient‐reported scales, and adverse effects were limited to transient local reactions. Importantly, ultrasonographic analysis demonstrated reductions exceeding 25% in submental fat thickness in most patients, suggesting substantial remodeling of adipose tissue architecture. Additional evidence comes from a large retrospective study involving 343 patients treated over a five‐year period with combinations of recombinant collagenase, hyaluronidase, and lipase for facial and body remodeling. Significant reductions in submental fat, abdominal circumference, cellulite severity, and localized adiposity were reported, accompanied by improvements in skin firmness and tissue quality. Notably, these outcomes were achieved using minimally invasive protocols with favorable tolerability profiles. The distinction between adipocytolysis and adipometabolic remodeling may have important clinical implications. Whereas adipocytolytic approaches inevitably generate local inflammation secondary to cellular destruction, lipase‐mediated remodeling may theoretically preserve tissue architecture while selectively reducing lipid content. Such an approach aligns more closely with the principles of regenerative and precision medicine, emphasizing functional optimization rather than tissue injury.

Although further mechanistic studies are necessary, the available evidence supports the inclusion of lipases within the EB category and suggests that enzymatic modulation of adipose tissue may represent a new therapeutic paradigm for localized fat reduction and body contouring.

### 3.6 Lyases and Extracellular Matrix Reorganization

While hyaluronidases and collagenases target specific extracellular matrix components, lyases contribute to tissue remodeling by broadly modulating glycosaminoglycans and proteoglycan‐rich microenvironments.

The extracellular matrix is composed not only of collagen fibers and hyaluronic acid but also of a complex network of proteoglycans and glycosaminoglycans that regulate hydration, growth factor signaling, angiogenesis, inflammation, and cellular communication [1, 2, 53, 54, 55, 56, 57]. Alterations in these structures are frequently associated with fibrosis, edema, cellulite, tissue aging, and impaired regenerative responses.

Lyases catalyze the cleavage of glycosaminoglycans via elimination reactions, yielding biochemical modifications distinct from those generated by classical hydrolases. Through selective degradation of matrix polysaccharides, these enzymes may reduce matrix density, improve tissue permeability, facilitate molecular diffusion, and promote remodeling of extracellular matrix architecture [53, 54, 55, 56].

The biological significance of this mechanism becomes particularly relevant in conditions characterized by extracellular matrix densification and impaired tissue dynamics. Cellulite, for example, is increasingly understood as a multifactorial disorder involving adipose tissue expansion, fibrosis, microcirculatory dysfunction, and extracellular matrix remodeling. Histological studies have demonstrated that cellulite is associated with thickened fibrous septa, altered collagen organization, and accumulation of extracellular matrix components, all of which contribute to the characteristic “orange peel” appearance.

A recent prospective study evaluating a combined enzymatic therapy consisting of lyase, lipase, and collagenase reported clinical improvement in cellulite severity following three treatment sessions. The authors proposed that the synergistic action of these enzymes simultaneously targeted the edematous, adipose, and fibrotic components of cellulite, thereby addressing multiple pathogenic mechanisms rather than a single anatomical structure.

This concept of simultaneous multi‐substrate remodeling is particularly relevant to the EB framework. Unlike therapies directed toward isolated biological targets, EBs may act synergistically on different components of the tissue microenvironment, facilitating broader structural reorganization while maintaining substrate specificity.

### 3.7 Keratinases and Tissue Renewal

Keratinases are the most exploratory component of the EB platform and illustrate the remarkable versatility of enzyme‐mediated tissue remodeling. These proteolytic enzymes possess the unique ability to degrade highly resistant keratinized structures, including proteins stabilized by extensive disulfide bonds [24, 25].

Traditionally, keratinases have been investigated primarily in biotechnology and industrial applications. However, recent evidence suggests that their biological effects may be relevant to regenerative medicine and tissue renewal. Recombinant keratinases have been shown to increase skin permeability and enhance the penetration of active compounds, creating opportunities for both therapeutic and cosmetic applications.

Additionally, emerging evidence suggests that recombinant keratinase‐based formulations may improve skin quality, hydration, elasticity, and the appearance of post‐acne scars when integrated into broader regenerative treatment protocols.

Although their role in aesthetic medicine remains less established than that of hyaluronidases, collagenases, and lipases, keratinases reinforce the central premise of the EB concept: selective catalytic modulation of biological substrates can be harnessed to promote structural and functional tissue remodeling.

Collectively, lipases, lyases, and keratinases demonstrate that the concept of EBs extends far beyond extracellular matrix degradation alone. Instead, it encompasses a versatile platform of substrate‐specific biological interventions that target adipose tissue, extracellular matrix components, proteoglycans, and keratinized structures via precise catalytic mechanisms. This versatility represents one of the most distinctive features of EBs and supports their recognition as a novel therapeutic category in aesthetic and regenerative medicine (Table 2).

**TABLE 2:** Summary of the principal clinical studies supporting Enzymatic Bioremodelers (EBs).

First Author (Year)	Clinical indication	Recombinant enzyme(s)	Study design	Treatment protocol	Treatment interval	Main findings	Clinical relevance
de Castro García and Muñoz Montaño (2020) [37]	Hypertrophic, atrophic and keloid scars	Collagenase PB220 + Lipase PB500 + Lyase PB72K + HMW‐HA	Prospective multicenter study (42 patients; 44 scars)	Three intralesional treatment sessions	21 days	Significant improvement in Vancouver and POSAS scores, pigmentation, vascularity, pliability, and scar relief	Demonstrates enzymatic remodeling of pathological scars through extracellular matrix modulation
Giraldo Mesa et al. (2025) [38]	Post‐liposuction fibrosis	Collagenase PB220 + Lipase PB500 + Lyase PB72K + HMW‐HA	Clinical and histological study	Single treatment session	—	Histological reduction of fibrosis and inflammatory infiltrate, improved collagen organization, restoration of elastic fibers, and improved skin architecture	Provides histological evidence supporting regenerative extracellular matrix remodeling
Jabbour et al. (2024) [39]	Moderate‐to‐severe submental fat	Lipase PB500 + Collagenase PB220 + Hyaluronidase PB3000 + HMW‐HA	Prospective clinical study	Three treatment sessions	21 days	Significant reduction in submental fat thickness by ultrasound, high physician and patient satisfaction	Supports adipometabolic remodeling without direct adipocytolysis
Castelanich et al. (2024) [40]	Late‐onset periocular edema after HA fillers	Hyaluronidase PB3000 + Collagenase PB220 + HMW‐HA	Retrospective case series (6 patients)	Single treatment session	—	Complete edema resolution without post‐hyaluronidase syndrome or significant adverse events	Demonstrates physiological extracellular matrix normalization in filler‐related complications
Castelanich et al. (2025) [41]	Foreign body granulomas	Collagenase PB220 + Hyaluronidase PB3000 + Lipase PB500 + HMW‐HA	Case reports	Three treatment sessions	21 days	Clinical, ultrasonographic, and histological resolution of granulomatous lesions	Supports multimodal enzymatic remodeling of chronic inflammatory lesions
Wu (2025) [42]	Polycaprolactone (Ellansé) nodules	Collagenase‐based formulation	Case series and experimental rheological study	Individualized according to lesion response	Approximately 21–30 days*	Complete clinical resolution of persistent nodules and rheological degradation of polycaprolactone	Establishes collagenase as a potential enzymatic reversal strategy for collagen stimulator complications
Rivera et al. (2025) [43]	Facial and body remodeling	Collagenase PB220 + Lipase PB500 + Hyaluronidase PB3000 + Lyase PB72K + HMW‐HA	Retrospective multicenter study (343 patients)	Individualized according to indication	21–30 days	Improvement in localized adiposity, cellulite, facial contour, skin quality, and tissue firmness	Demonstrates broad clinical applicability of recombinant enzymatic remodeling
Castelanich et al. (2024) [72]	Epidermoid (sebaceous) cyst	Collagenase PB220 + Hyaluronidase PB3000 + Lipase PB500 + HMW‐HA	Case report	Single intralesional treatment	—	Complete clinical and ultrasonographic resolution without surgical excision	Provides preliminary evidence supporting recombinant enzymatic therapy as a minimally invasive alternative for selected epidermoid cysts

*Note:* The recombinant enzymes discussed in this review include Hyaluronidase PB3000 ( *Streptococcus pyogenes* recombinant origin), Collagenase G/H PB220 ( *Clostridium histolyticum* recombinant origin), Lipase PB500 ( *Thermus thermophilus* recombinant origin), and Lyase PB72K, developed by Proteos Biotech S.L. (Albacete, Spain). These enzymes are produced using recombinant protein engineering technologies and are characterized by high purity, substrate specificity, and controlled catalytic activity. Published clinical studies have consistently employed treatment intervals of approximately 21–30 days, a schedule supported primarily by biological tissue remodeling, allowing inflammatory resolution, extracellular matrix reorganization, collagen turnover, and tissue stabilization before subsequent enzymatic intervention, although comparative pharmacokinetic studies remain unavailable. *Treatment intervals marked with* (*) were not explicitly standardized in the original publication and were inferred from the reported treatment protocol.* Source: Elaborated by the author.

Abbreviations: ECM, matriz extracelular, extracellular matrix; EBs, Enzymatic Bioremodelers; HA, hyaluronic acid; HMW‐HA, high‐molecular‐weight hyaluronic acid; PB, recombinant bacterial enzyme.

### 3.8 Complication Management With Enzymatic Bioremodelers

One of the most promising and rapidly expanding applications of applications of EBs lies in the management of aesthetic complications. Traditionally, adverse events resulting from fillers, collagen stimulators, foreign materials, fibrosis, and inflammatory reactions have been managed using corticosteroids, antibiotics, surgical excision, drainage procedures, or observation alone. Although these approaches may yield satisfactory outcomes in selected cases, they often fail to address the underlying biological substrate of the complication. In contrast, EBs offer a mechanism‐based therapeutic strategy that directly targets the structural and extracellular matrix alterations that sustain pathological tissue responses.

The management of hyaluronic acid (HA)‐related complications is among the earliest and most established examples of enzyme‐based intervention in aesthetic medicine. Hyaluronidase has long been considered the gold standard for treating vascular compromise, nodules, overcorrection, Tyndall effect, and delayed edema associated with HA fillers [11, 12, 58]. By selectively hydrolyzing hyaluronic acid chains, hyaluronidase promotes the degradation of both exogenous and endogenous HA, thereby facilitating the restoration of tissue homeostasis.

However, recent observations have highlighted a potential limitation of repeated or excessive hyaluronidase administration. Castelanich et al. described the phenomenon termed *post‐hyaluronidase syndrome*, characterized by tissue hollowing, loss of volume, skin atrophy, reduced elasticity, and pigmentation changes following extensive enzymatic degradation of the extracellular matrix [40].

To address this limitation, the same group proposed a novel therapeutic approach that combines high‐dose hyaluronidase with low‐dose collagenase to treat late‐onset periocular edema following HA filler injections. In a retrospective review involving six patients, all subjects demonstrated significant clinical improvement after a single treatment session, with no evidence of post‐hyaluronidase syndrome or relevant adverse effects. Improvement was confirmed using validated aesthetic scales, and no additional treatments were required during follow‐up. These findings suggest that combined enzymatic modulation may promote a more physiological remodeling of the extracellular matrix than isolated degradation of hyaluronic acid alone [40].

Foreign body granulomas constitute another important area in which EBs may offer unique therapeutic advantages. Granulomatous reactions arise when the immune system fails to eliminate exogenous materials, leading to persistent inflammation, macrophage aggregation, fibrosis, and extracellular matrix remodeling. Conventional therapies include intralesional corticosteroids, topical immunomodulators, laser treatments, and surgical excision, although outcomes are often unpredictable and recurrence remains common.

Castelanich et al. reported two cases of foreign body granulomas successfully treated with a combination of recombinant collagenase, hyaluronidase, and lipase. Following three treatment sessions, both patients exhibited marked clinical improvement without adverse events. Histological analysis in one patient demonstrated complete resolution of granulomatous tissue, while ultrasonographic follow‐up in the second case confirmed a substantial reduction in lesion size [41].

The biological rationale for this approach is particularly compelling. Granulomas frequently contain significant fibrotic and extracellular matrix components in addition to inflammatory infiltrates. Therefore, simultaneous modulation of collagen, hyaluronic acid, adipose tissue remnants, and extracellular matrix organization may explain the favorable clinical outcomes observed. These cases illustrate how EBs can function not only as aesthetic remodeling tools but also as therapeutic agents capable of resolving chronic pathological tissue responses.

A similar principle has recently been applied to complications associated with collagen stimulators. Polycaprolactone‐based fillers, such as Ellansé, have gained popularity for their ability to induce neocollagenesis and provide long‐lasting tissue rejuvenation. Nevertheless, delayed‐onset nodules remain one of their most challenging complications due to the lack of a specific reversal agent comparable to hyaluronidase, which is available for HA fillers.

In a recent case series, Wu described the successful treatment of 10 persistent Ellansé‐associated nodules in 3 patients using a collagenase‐based protocol known as the *iCare Technique*. Clinical resolution was achieved in all cases, and experimental analyses demonstrated that collagenase mixtures altered the rheological properties of the polycaprolactone‐based material, transforming it from a gel‐like structure into a fluid solution within minutes [42].

The implications of these findings are substantial. For the first time, collagenase emerges as a potential enzymatic reversal strategy for complications associated with biostimulatory fillers. This observation expands the scope of EBs beyond tissue remodeling and positions them as precision tools capable of managing complications previously considered difficult or impossible to reverse through minimally invasive methods.

Beyond fillers and granulomas, EBs may also contribute to the management of fibrosis‐related complications. Fibrosis is a common pathological endpoint shared by multiple aesthetic complications, including post‐liposuction irregularities, scar contractures, filler‐induced fibrosis, and chronic inflammatory reactions.

Histological studies evaluating recombinant enzymatic combinations in post‐liposuction fibrosis demonstrated reductions in inflammatory infiltrates, improved collagen organization, restoration of elastic fibers, and overall improvement of tissue architecture [38]. Similarly, multicenter studies involving pathological scars demonstrated significant improvements in pigmentation, vascularity, pliability, relief, and patient‐reported outcomes after treatment with recombinant collagenase‐, lipase‐, and lyase‐based formulations combined with hyaluronic acid [37].

Taken together, the available evidence indicates that EBs represent a novel therapeutic platform for complication management in aesthetic medicine. Unlike conventional approaches that primarily suppress inflammation or physically remove pathological tissue, EBs directly target the structural substrates responsible for tissue dysfunction. Through selective modulation of collagen, hyaluronic acid, adipose tissue components, and extracellular matrix architecture, these enzymes provide a mechanistically based strategy to restore tissue homeostasis while minimizing collateral damage.

This expanding body of evidence suggests that the future role of EBs may extend beyond elective aesthetic enhancement. Their ability to simultaneously address fibrosis, inflammation, extracellular matrix disorganization, and pathological tissue remodeling positions them as versatile therapeutic agents with applications in both corrective and regenerative medicine. As additional clinical studies emerge, EBs may become an integral component of complication management algorithms and a cornerstone of precision regenerative aesthetics.

### 3.9 Enzymatic Bioremodelers Versus Conventional Tissue Remodeling Therapies

One of the strongest arguments for recognizing EBs as a distinct therapeutic category is the fundamental difference between their mechanism of action and that of conventional tissue‐remodeling therapies currently employed in aesthetic and regenerative medicine. Although many therapeutic modalities ultimately aim for similar clinical outcomes, such as fibrosis reduction, scar improvement, adipose tissue remodeling, tissue rejuvenation, and restoration of structural support, the biological pathways by which these outcomes are achieved differ substantially.

Among the most widely used therapies in contemporary aesthetic practice are injectable collagen stimulators, including poly‐L‐lactic acid (PLLA), calcium hydroxylapatite (CaHA), and polycaprolactone (PCL). These biomaterials induce tissue remodeling primarily through a controlled foreign‐body response that stimulates fibroblast activation, extracellular matrix turnover, and subsequent neocollagenesis [59, 60, 61, 62]. Their efficacy has been extensively demonstrated for the treatment of volume loss, skin laxity, and age‐related structural deterioration. However, the biological effects of these materials depend largely on the induction of a secondary inflammatory response that subsequently triggers regenerative processes.

In contrast, EBs do not require an intermediary inflammatory stimulus to initiate tissue remodeling. Their activity is mediated through direct catalytic interaction with specific molecular substrates present within the tissue microenvironment. Hyaluronidases selectively hydrolyze hyaluronic acid, collagenases degrade pathological collagen accumulations, lipases catalyze triglyceride hydrolysis, and lyases modify glycosaminoglycans and proteoglycan‐rich matrix structures. Consequently, EBs achieve remodeling through substrate‐specific biochemical modulation rather than through inflammation‐dependent regenerative cascades.

Another important distinction can be observed when comparing EBs with adipocytolytic agents such as deoxycholic acid. Injectable deoxycholic acid induces localized fat reduction via detergent‐mediated disruption of adipocyte membranes, resulting in cellular lysis, inflammatory infiltration, and subsequent tissue remodeling [17, 18]. Although highly effective for reducing submental fullness and localized adiposity, this mechanism is intrinsically dependent on tissue injury and inflammatory clearance of cellular debris.

Recent studies evaluating combinations of recombinant enzymes containing lipase, collagenase, and hyaluronidase suggest an alternative biological paradigm. Clinical improvements in localized adiposity, submental fat, body contouring, and cellulite have been observed following enzymatic treatment without relying primarily on adipocyte destruction [39, 43]. These findings support the concept of adipometabolic remodeling, whereby tissue architecture is modified through selective hydrolysis of lipid substrates and modulation of adipose tissue physiology rather than through direct cytolysis. This distinction may have important implications for tissue preservation, inflammatory burden, and long‐term regenerative outcomes.

Mechanical remodeling procedures provide another useful comparison. Techniques such as subcision and microneedling rely on controlled physical injury to initiate wound healing responses, fibroblast recruitment, and collagen synthesis [63, 64]. Their efficacy is directly linked to the body’s capacity to respond to mechanical trauma through inflammation and tissue repair. Although highly effective in selected indications, these procedures fundamentally depend on tissue disruption as the initiating biological event.

EBs differ substantially from this approach. Rather than inducing mechanical injury, they interact directly with structural molecules that contribute to pathological tissue architecture. Remodeling, therefore, occurs through selective biochemical modification of existing tissue components, allowing targeted intervention without the need for controlled trauma.

A similar distinction exists when comparing EBs with intralesional corticosteroid therapies, which are frequently used in the management of hypertrophic scars, keloids, granulomas, and fibrosis. Corticosteroids exert their therapeutic effects primarily through suppression of inflammation, inhibition of fibroblast proliferation, and reduction of collagen synthesis [65, 66]. While clinically valuable, they do not directly modify the extracellular matrix structures responsible for tissue rigidity, fibrosis, or abnormal architecture.

In contrast, collagenases directly interact with pathological collagen deposits, while hyaluronidases, lipases, and lyases selectively target additional structural components of the tissue microenvironment. Clinical studies involving pathological scars, post‐liposuction fibrosis, foreign body granulomas, and filler‐related complications have demonstrated that recombinant enzymatic therapies can induce measurable structural remodeling, including improved collagen organization, restoration of elastic fibers, reduction of fibrosis, and normalization of extracellular matrix architecture [37, 38, 41].

The emerging evidence from complication management further highlights the uniqueness of EBs. Recent studies have demonstrated successful treatment of periocular edema following hyaluronic acid fillers, foreign body granulomas, and persistent nodules associated with polycaprolactone‐based collagen stimulators through selective enzymatic modulation of pathological tissue substrates [40, 41, 42]. These observations illustrate that EBs can function simultaneously as regenerative agents, remodeling therapies, and complication‐management tools, a versatility that is rarely observed among conventional aesthetic interventions.

Taken together, these comparisons reveal a defining characteristic that distinguishes EBs from most established tissue remodeling therapies. Whereas conventional approaches commonly rely on inflammation, mechanical injury, cellular destruction, or indirect biological stimulation, EBs operate through direct catalytic modulation of specific molecular targets. This mechanistic specificity provides a biologically grounded rationale for recognizing EBs as a unique therapeutic platform within aesthetic and regenerative medicine, positioned at the intersection of extracellular matrix biology, regenerative therapeutics, and precision medicine (Table 3).

**TABLE 3:** Comparison between enzymatic bioremodelers and conventional therapies.

Therapy	Primary mechanism	Tissue injury	Specificity	Remodeling
EBs	Catalytic modulation	Low	High	Direct
PLLA	Inflammation	Moderate	Moderate	Indirect
CaHA	Inflammation	Moderate	Moderate	Indirect
PCL	Inflammation	Moderate	Moderate	Indirect
Deoxycholic acid	Cytolysis	High	Low	Indirect
Subcision	Mechanical injury	High	Low	Indirect
Microneedling	Controlled injury	Moderate	Low	Indirect

*Note:* Elaborated by the author.

### 3.10 Safety Considerations, Limitations, and Translational Challenges

Despite the promising clinical and biological evidence supporting EBs, several challenges must be addressed before their widespread incorporation into evidence‐based therapeutic protocols. As with any emerging therapeutic platform, the long‐term success of EBs will depend not only on clinical efficacy but also on safety, reproducibility, regulatory validation, and mechanistic understanding.

One of the primary concerns associated with enzyme‐based therapies is immunogenicity. Therapeutic enzymes are proteins and, therefore, may potentially trigger immune responses depending on their origin, purity, formulation, and route of administration [67, 68]. Historically, immunogenic reactions have represented a major limitation for several protein‐based therapeutics. However, advances in recombinant biotechnology have substantially reduced this risk through improved purification techniques, elimination of contaminating proteins, and optimization of molecular structure [7, 67]. The recombinant enzymes currently evaluated in aesthetic medicine are produced under controlled biotechnological conditions and have generally demonstrated favorable safety profiles in clinical studies [37, 38, 39, 40, 41].

An additional aspect deserving consideration is the potential development of antibodies against collagenase following repeated exposure. Clinical studies evaluating collagenase *C. histolyticum* for Dupuytren's contracture and Peyronie's disease have demonstrated that most treated patients develop binding antibodies against collagenase Class I and II after repeated injections. Importantly, despite the high seroconversion rates, these antibodies have not been associated with reduced clinical efficacy, neutralizing activity, hypersensitivity reactions, or cumulative toxicity. Current evidence therefore suggests that antibody formation represents an expected immunological response to repeated exposure to a bacterial protein rather than a clinically relevant safety concern. Nevertheless, because recombinant collagenases have only recently been introduced into aesthetic and regenerative medicine, long‐term surveillance studies remain necessary to determine whether similar immunological profiles will be observed following repeated cosmetic treatments [14, 58].

Clinical evidence published to date has consistently reported a low incidence of serious adverse events. In studies involving scar remodeling, cellulite treatment, submental fat reduction, post‐liposuction fibrosis, and facial reshaping, adverse effects were predominantly mild and transient, including local edema, discomfort, ecchymosis, and transient inflammatory reactions [37, 38, 39, 43]. Similarly, studies evaluating the treatment of granulomas, periocular edema, and filler‐related complications reported favorable tolerability profiles, with no severe complications or long‐term sequelae [40, 41].

Another important consideration relates to tissue specificity and enzymatic diffusion. Because EBs exert their effects through catalytic activity, their therapeutic success depends on adequate localization within the target tissue. Excessive diffusion may theoretically affect adjacent structures, whereas insufficient diffusion may limit efficacy. This issue is particularly relevant for enzymes targeting extracellular matrix components, where precise delivery is essential to achieve predictable outcomes. Future advances in delivery systems, including biomaterials, injectable hydrogels, and nanotechnology‐based carriers, may improve tissue targeting and optimize pharmacokinetic behavior [38].

A further challenge concerns the heterogeneity of available evidence. While hyaluronidase and collagenase possess well‐established biological mechanisms and decades of clinical use, other enzymes, such as lipases, lyases, and keratinases, remain in earlier stages of clinical translation. Although emerging studies have demonstrated promising results in adipose tissue remodeling, cellulite, fibrosis, and skin quality enhancement, larger randomized controlled trials are still needed to confirm efficacy and establish standardized treatment protocols [39, 43].

The concept of adipometabolic remodeling illustrates this challenge particularly well. Current evidence suggests that recombinant lipases may reduce localized adiposity and improve tissue contour without directly destroying adipocytes [39, 43]. However, the precise biological mechanisms responsible for these outcomes remain incompletely understood. Additional studies investigating adipose tissue histology, inflammatory responses, metabolic changes, and long‐term tissue behavior will be necessary to clarify the role of lipases within the EB framework.

The same consideration applies to keratinases and matrix‐targeting lyases. Although their biological rationale is supported by robust biochemical evidence and preliminary clinical observations, large‐scale clinical investigations remain limited. Consequently, these enzymes should currently be viewed as emerging components of the EB platform whose full therapeutic potential remains to be established.

From a regulatory perspective, another important challenge involves classification. Many enzyme‐based products currently occupy a hybrid space between medical devices, biologics, and therapeutic proteins. This regulatory complexity may vary among jurisdictions and influence clinical adoption, post‐marketing surveillance, and future product development. The establishment of EBs as a distinct conceptual category may facilitate future regulatory discussions by emphasizing their shared biological mechanism rather than isolated clinical indications.

Several limitations of the present review should also be acknowledged. First, as a narrative review, the study does not synthesize quantitative evidence and is inherently subject to selection bias. Second, the clinical evidence available for certain enzyme classes remains limited compared with that for more established therapeutic modalities. Third, many of the studies reviewed involve relatively small patient populations, observational designs, or case‐series methodologies. Consequently, portions of the proposed framework should be interpreted as translational and hypothesis‐generating rather than definitive clinical guidance.

Nevertheless, these limitations do not diminish the conceptual relevance of the findings. On the contrary, they highlight the need for further research and underscore the importance of establishing a coherent scientific framework to guide future investigations. The convergence of extracellular matrix biology, regenerative medicine, recombinant protein engineering, and clinical aesthetics provides a strong biological foundation for the continued development of EBs.

As the field advances, future studies should focus on randomized clinical trials, mechanistic histological investigations, biomarker analyses, imaging‐based outcome assessments, and long‐term safety evaluations. Such efforts will be essential for defining optimal treatment protocols, identifying new therapeutic targets, and further validating EBs as a distinct platform for precision tissue remodeling.

### 3.11 Future Perspectives

The convergence of biotechnology, regenerative medicine, extracellular matrix biology, and protein engineering is creating unprecedented opportunities for the development of highly targeted therapeutic interventions. Within this context, EBs represent a promising platform capable of redefining how tissue remodeling is approached in both aesthetic and regenerative medicine. The concept proposed in this review extends beyond the currently available enzymes and establishes a framework that may accommodate future generations of recombinant proteins specifically engineered to interact with selected biological substrates.

Recent advances in recombinant DNA technology, protein engineering, and directed evolution have transformed therapeutic enzymes from niche biological tools into sophisticated precision therapeutics [7, 68, 69]. Modern recombinant proteins can be designed to exhibit enhanced substrate specificity, improved catalytic efficiency, greater stability, lower immunogenicity, and optimized pharmacokinetic profiles. These technological developments are particularly relevant for EBs because their therapeutic effects are intrinsically linked to substrate recognition and catalytic precision.

Future generations of EBs may be developed to selectively target specific extracellular matrix components, fibrotic structures, adipose tissue compartments, inflammatory microenvironments, or pathological protein aggregates. Such an approach would allow clinicians to intervene directly in the biological mechanisms underlying tissue dysfunction rather than relying on indirect stimulation or on generalized tissue injury. This strategy is highly consistent with the principles of precision medicine, which seek to individualize treatment based on biological targets and mechanistic understanding rather than solely on clinical presentation [70, 71].

The integration of advanced delivery systems may further enhance the therapeutic potential of EBs. Biomaterials, injectable hydrogels, controlled‐release platforms, and nanotechnology‐based carriers are increasingly being investigated as methods to improve protein stability, tissue retention, and target specificity [38]. Such technologies may reduce off‐target effects while maximizing local enzymatic activity, thereby improving both safety and efficacy.

Another promising avenue involves combining EBs with established regenerative therapies. The extracellular matrix is increasingly recognized as a central regulator of cellular behavior, stem cell activity, angiogenesis, and tissue repair [1, 2]. Selective enzymatic remodeling of the tissue microenvironment may therefore create conditions that enhance the effectiveness of collagen stimulators, platelet‐derived products, stem cell therapies, biomaterials, and tissue engineering strategies. Rather than functioning as isolated interventions, EBs may become important components of integrated regenerative protocols.

The expanding clinical evidence reviewed in this article also suggests that the future role of EBs may extend far beyond traditional aesthetic applications. Fibrosis, chronic wounds, scar contractures, granulomatous disorders, foreign body reactions, connective tissue diseases, and postoperative complications all represent potential therapeutic targets for selective enzymatic remodeling [37, 38, 40, 41]. In this regard, the concept of EBs may ultimately contribute to the development of a broader regenerative therapeutic paradigm that transcends the boundaries between aesthetic medicine, dermatology, plastic surgery, and tissue engineering.

Although the clinical evidence remains extremely limited, recombinant enzymatic therapy has recently been reported as a minimally invasive alternative for the treatment of epidermoid (sebaceous) cysts, with complete clinical resolution documented in a single case report using a combination of recombinant collagenase, hyaluronidase, and lipase. While this observation expands the potential spectrum of enzyme‐based therapies, this indication should currently be considered investigational until validated by larger prospective studies [72].

As clinical experience continues to grow, future investigations should prioritize randomized controlled trials, histological analyses, molecular biomarker studies, and long‐term outcome evaluations. Such research will be essential not only for validating individual enzyme applications but also for refining the broader conceptual framework proposed in this review. Ultimately, the future of EBs lies in their ability to integrate biological specificity, catalytic precision, and regenerative potential into a unified therapeutic platform that promotes targeted tissue remodeling with minimal collateral damage.

## 4 Conclusion

The concept of EBs offers a new perspective on the role of therapeutic enzymes in aesthetic and regenerative medicine. Although different enzymes act on distinct biological substrates, they share a common mechanism of selective catalytic activity that promotes targeted tissue remodeling and restoration of tissue homeostasis.

Current evidence suggests that EBs have broad therapeutic potential, ranging from the treatment of fibrosis, scars, localized adiposity, and cellulite to the management of aesthetic complications and the improvement of tissue quality. Their ability to interact directly with the structural and functional components of the tissue microenvironment distinguishes them from conventional therapies that primarily rely on inflammation, mechanical disruption, or tissue destruction. Future investigations should focus on standardized treatment protocols, optimal dosing intervals, long‐term immunogenicity, and randomized controlled trials comparing recombinant enzymatic therapies with established aesthetic interventions. Such evidence will be essential to consolidate EBs as a precision‐based therapeutic platform within regenerative aesthetics.

By establishing a biologically grounded, mechanism‐based classification, the EB framework provides a foundation for integrating existing enzyme‐based therapies and supporting the development of future recombinant technologies and precision regenerative interventions. As knowledge in extracellular matrix biology, protein engineering, and regenerative medicine continues to advance, EBs may emerge as an important therapeutic platform for targeted, minimally invasive, and biologically guided tissue remodeling.

## Author Contributions

Antony de Paula Barbosa conceived the study, developed the conceptual framework, performed the literature review, interpreted the data, drafted the manuscript, critically revised the content, and approved the final version of the manuscript.

## Funding

The author has nothing to report.

## Ethics Statement

The author has nothing to report.

## Conflicts of Interest

Antony de Paula Barbosa serves as a speaker and consultant for Galderma Aesthetics Brazil, Blue Medical, and Welt Medical. Blue Medical and Welt Medical are distributors of Proteos Biotech (Spain) in Brazil. The author declares that these affiliations did not influence the content of this manuscript and reports no conflicts of interest related to the subject matter presented.

## Data Availability

Data sharing is not applicable to this article, as no new data were created or analyzed in this study.

## References

[1] C. Frantz , K. M. Stewart , and V. M. Weaver , “The Extracellular Matrix at a Glance,” Journal of Cell Science 123, no. 24 (2010): 4195–4200, 10.1242/jcs.023820. PMC2995612 21123617

[2] A. D. Theocharis , S. S. Skandalis , C. Gialeli , and N. K. Karamanos , “Extracellular matrix structure,” Advanced Drug Delivery Reviews 97 (2016): 4–27, 10.1016/j.addr.2015.11.001. 26562801

[3] T. A. Wynn , “Cellular and Molecular Mechanisms of Fibrosis,” Journal of Pathology 214, no. 2 (2008): 199–210, 10.1002/path.2277. PMC2693329 18161745

[4] C. Bonnans , J. Chou , and Z. Werb , “Remodelling the Extracellular Matrix in Development and Disease,” Nature Reviews. Molecular Cell Biology 15, no. 12 (2014): 786–801, 10.1038/nrm3904. PMC4316204 25415508

[5] D. E. Discher , P. Janmey , and Y. L. Wang , “Tissue Cells Feel and Respond to the Stiffness of Their Substrate,” Science 310, no. 5751 (2005): 1139–1143, 10.1126/science.1116995. 16293750

[6] E. E. Kershaw and J. S. Flier , “Adipose Tissue as an Endocrine Organ,” Journal of Clinical Endocrinology and Metabolism 89, no. 6 (2004): 2548–2556, 10.1210/jc.2004-0395. 15181022

[7] G. Walsh , “Biopharmaceutical Benchmarks 2018,” Nature Biotechnology 36, no. 12 (2018): 1136–1145, 10.1038/nbt.4305. 30520869

[8] B. Leader , Q. J. Baca , and D. E. Golan , “Protein Therapeutics: A Summary and Pharmacological Classification,” Nature Reviews. Drug Discovery 7, no. 1 (2008): 21–39, 10.1038/nrd2399. 18097458

[9] C. DeLorenzi , “Complications of Injectable Fillers, Part 2: Vascular Complications,” Aesthetic Surgery Journal 34, no. 4 (2014): 584–600, 10.1177/1090820X14525035. 24692598

[10] A. Fallacara , E. Baldini , S. Manfredini , and S. Vertuani , “Hyaluronic Acid in the Third Millennium,” Polymers 10, no. 7 (2018): 701, 10.3390/polym10070701. PMC6403654 30960626

[11] R. Stern and M. J. Jedrzejas , “Hyaluronidases and Their Genomics,” Chemical Reviews 106, no. 3 (2006): 818–839, 10.1021/cr050247k. PMC2547145 16522010

[12] K. Beleznay , J. D. A. Carruthers , S. Humphrey , and D. Jones , “Vascular Compromise From Soft Tissue Augmentation: Experience With 12 Cases and Recommendations for Optimal Outcomes,” Dermatologic Surgery 41, no. 10 (2015): 1097–1117, 10.1097/DSS.0000000000000509. PMC4174919 25276276

[13] L. C. Hurst , M. A. Badalamente , V. R. Hentz , et al., “Injectable Collagenase *clostridium histolyticum* for Dupuytren's Contracture,” New England Journal of Medicine 361, no. 10 (2009): 968–979, 10.1056/NEJMoa0810866. 19726771

[14] M. Gelbard , I. Goldstein , W. J. G. Hellstrom , et al., “Clinical Efficacy, Safety and Tolerability of Collagenase *clostridium histolyticum* for Peyronie's Disease,” Journal of Urology 190, no. 1 (2013): 199–207, 10.1016/j.juro.2013.01.087. 23376148

[15] D. Langin , “Adipose Tissue Lipolysis as a Metabolic Pathway to Define Pharmacological Strategies Against Obesity and the Metabolic Syndrome,” Pharmacological Research 53, no. 6 (2006): 482–491, 10.1016/j.phrs.2006.03.009. 16644234

[16] R. Zechner , P. C. Kienesberger , G. Haemmerle , R. Zimmermann , and A. Lass , “Adipose Triglyceride Lipase and the Lipolytic Catabolism of Cellular Fat Stores,” Journal of Lipid Research 50, no. 1 (2009): 3–21, 10.1194/jlr.R800031-JLR200. 18952573

[17] E. D. Deeks , “Deoxycholic Acid: A Review in Submental Fat Contouring,” American Journal of Clinical Dermatology 17, no. 6 (2016): 701–707, 10.1007/s40257-016-0231-3. 27785706

[18] A. M. Rotunda , H. Suzuki , R. L. Moy , and M. S. Kolodney , “Detergent Effects of Sodium Deoxycholate Are a Major Feature of an Injectable Phosphatidylcholine Formulation Used for Localized Fat Dissolution,” Dermatologic Surgery 30, no. 7 (2004): 1001–1008, 10.1111/j.1524-4725.2004.30305.x. 15209790

[19] M. Lafontan and D. Langin , “Lipolysis and Lipid Mobilization in Human Adipose Tissue,” Progress in Lipid Research 48, no. 5 (2009): 275–297, 10.1016/j.plipres.2009.05.001. 19464318

[20] P. Arner , “Human Fat Cell Lipolysis: Biochemistry, Regulation and Clinical Role,” Best Practice & Research. Clinical Endocrinology & Metabolism 19, no. 4 (2005): 471–482, 10.1016/j.beem.2005.07.004. 16311212

[21] R. Zechner , R. Zimmermann , T. O. Eichmann , et al., “FAT SIGNALS: Lipases and Lipolysis in Lipid Metabolism and Signaling,” Cell Metabolism 15, no. 3 (2012): 279–291, 10.1016/j.cmet.2011.12.018. PMC3314979 22405066

[22] E. D. Rosen and B. M. Spiegelman , “What We Talk About When We Talk About Fat,” Cell 156, no. 1–2 (2014): 20–44, 10.1016/j.cell.2013.12.012. PMC3934003 24439368

[23] K. Sun , C. M. Kusminski , and P. E. Scherer , “Adipose Tissue Remodeling and Obesity,” Journal of Clinical Investigation 121, no. 6 (2011): 2094–2101, 10.1172/JCI45887. PMC3104761 21633177

[24] R. Gupta and P. Ramnani , “Microbial Keratinases and Their Prospective Applications: An Overview,” Applied Microbiology and Biotechnology 70, no. 1 (2006): 21–33, 10.1007/s00253-005-0239-8. 16391926

[25] A. Brandelli , D. J. Daroit , and A. Riffel , “Biochemical Features of Microbial Keratinases and Their Production and Applications,” Applied Microbiology and Biotechnology 85, no. 6 (2010): 1735–1750, 10.1007/s00253-009-2398-5. 20039036

[26] G. C. Gurtner , S. Werner , Y. Barrandon , and M. T. Longaker , “Wound Repair and Regeneration,” Nature 453, no. 7193 (2008): 314–321, 10.1038/nature07039. 18480812

[27] S. A. Eming , P. Martin , and M. Tomic‐Canic , “Wound Repair and Regeneration: Mechanisms, Signaling and Translation,” Science Translational Medicine 6, no. 265 (2014): 265sr6, 10.1126/scitranslmed.3009337. PMC4973620 25473038

[28] P. Martin , “Wound Healing: Aiming for Perfect Skin Regeneration,” Science 276, no. 5309 (1997): 75–81, 10.1126/science.276.5309.75. 9082989

[29] A. J. Singer and R. A. F. Clark , “Cutaneous Wound Healing,” New England Journal of Medicine 341, no. 10 (1999): 738–746, 10.1056/NEJM199909023411006. 10471461

[30] T. A. Wynn and T. R. Ramalingam , “Mechanisms of Fibrosis: Therapeutic Translation for Fibrotic Disease,” Nature Medicine 18, no. 7 (2012): 1028–1040, 10.1038/nm.2807. PMC3405917 22772564

[31] F. Klingberg , B. Hinz , and E. S. White , “The Myofibroblast Matrix: Implications for Tissue Repair and Fibrosis,” Journal of Pathology 229, no. 2 (2013): 298–309, 10.1002/path.4104. PMC4005341 22996908

[32] B. Hinz , “Formation and Function of the Myofibroblast During Tissue Repair,” Journal of Investigative Dermatology 127, no. 3 (2007): 526–537, 10.1038/sj.jid.5700613. 17299435

[33] B. N. Green , C. D. Johnson , and A. Adams , “Writing Narrative Literature Reviews for Peer‐Reviewed Journals: Secrets of the Trade,” Journal of Chiropractic Medicine 5, no. 3 (2006): 101–117, 10.1016/S0899-3467(07)60142-6. PMC2647067 19674681

[34] R. Ferrari , “Writing Narrative Style Literature Reviews,” Medical Writing 24, no. 4 (2015): 230–235, 10.1179/2047480615Z.000000000329.

[35] C. Baethge , S. Goldbeck‐Wood , and S. Mertens , “SANRA: A Scale for the Quality Assessment of Narrative Review Articles,” Research Integrity and Peer Review 4 (2019): 5, 10.1186/s41073-019-0064-8. PMC6434870 30962953

[36] J. Ramundo and M. Gray , “Enzymatic Wound Debridement,” Journal of Wound, Ostomy, and Continence Nursing 35, no. 3 (2008): 273–280, 10.1097/01.WON.0000313611.78452.65. 18496083

[37] M. P. De Castro García and J. R. Muñoz Montaño , “Multicentre Clinical Study to Evaluate the Efficacy and Safety of Hyaluronic Acid and Enzyme Cocktail on Scars,” Dermatología CMQ 18, no. 2 (2020): 84–92.

[38] J. A. Giraldo Mesa , G. Matute Turizo , S. M. Vieira Ríos , V. Kopytina , and J. López Berroa , “Clinical and Histological Findings Following the Application of a Single Session of Recombinant Enzymes on the Face,” AboutOpen 13, no. 1 (2026): 1–6, 10.33393/ao.2026.3555.

[39] R. Jabbour , F. Farah , F. Mallat , et al., “Efficacy and Safety of the Enzymatic Mixture—Lipase, Collagenase and Hyaluronidase—In the Treatment of Moderate to Severe Submental Fat: A Prospective Cohort Study,” Heliyon 10 (2024): e25759, 10.1016/j.heliyon.2024.e25759. PMC10875420 38375282

[40] D. G. Castelanich , L. A. Parra Hernández , A. Martinez Amado , et al., “Combined Application of Hyaluronidase and Collagenase for Late‐Onset Edema in Periocular Area After Hyaluronic Acid Volume Repositioning: A Six‐Case Retrospective Review,” Cureus 16, no. 11 (2024): e74297, 10.7759/cureus.74297. PMC11665833 39717328

[41] D. G. Castelanich , D. Pascuzzi , and J. López Berroa , “Treatment of Foreign Body Granulomas With Combined Enzyme Therapy,” Acta Dermatovenerologica Alpina, Pannonica et Adriatica 34 (2025): 75–77, 10.15570/actaapa.2025.17. 40579961

[42] L. Wu , “iCare Technique of Dissolving Ellansé M Nodules Using Collagenase: A Case Series and Experimental Study,” Journal of Cosmetic Dermatology 24 (2025): e70201, 10.1111/jocd.70201. PMC12038313 40296530

[43] Z. Rivera , I. Rivera , D. Ortiz , D. A. Lugo , I. Hagel , and V. Ollarves , “Recombinant Enzyme Treatment: 5 Years of Experience in Face and Body Reshaping,” Medicina Estética 84, no. 3 (2025): 39–52, 10.48158/MEDICINAESTETICA.084.04.

[44] M. J. Jedrzejas , “Hyaluronan Lyases: Structure, Function, Mechanism of Action and Substrate Specificity,” Critical Reviews in Biochemistry and Molecular Biology 37, no. 5 (2002): 297–325, 10.1080/10409230290771543.

[45] D. Jiang , J. Liang , and P. W. Noble , “Hyaluronan as an Immune Regulator in Human Diseases,” Physiological Reviews 91, no. 1 (2011): 221–264, 10.1152/physrev.00052.2009. PMC3051404 21248167

[46] J. M. Cyphert , C. S. Trempus , and S. Garantziotis , “Size Matters: Molecular Weight Specificity of Hyaluronan Effects in Cell Biology,” International Journal of Cell Biology 2015 (2015): 563818, 10.1155/2015/563818. PMC4581549 26448754

[47] R. Stern , A. A. Asari , and K. N. Sugahara , “Hyaluronan Fragments: An Information‐Rich System,” European Journal of Cell Biology 85, no. 8 (2006): 699–715, 10.1016/j.ejcb.2006.05.009. 16822580

[48] S. N. A. Bukhari , N. L. Roswandi , M. Waqas , et al., “Hyaluronic Acid, a Promising Skin Rejuvenating Biomedicine: A Review of Recent Updates and Pre‐Clinical and Clinical Investigations on Cosmetic and Nutricosmetic Effects,” International Journal of Biological Macromolecules 120 (2018): 1682–1695, 10.1016/j.ijbiomac.2018.09.188. 30287361

[49] P. J. McKeown‐Longo and P. J. Higgins , “Hyaluronan, Transforming Growth Factor β, and Extra Domain A‐Fibronectin: A Fibrotic Triad,” Advances in Wound Care (New Rochelle) 10, no. 3 (2021): 137–149, 10.1089/wound.2020.1192. PMC7876357 32667849

[50] S. M. Ruppert , T. R. Hawn , A. Arrigoni , T. N. Wight , and P. L. Bollyky , “Tissue Integrity Signals Communicated by High‐Molecular Weight Hyaluronan and the Resolution of Inflammation,” Immunologic Research 58, no. 2–3 (2014): 186–192, 10.1007/s12026-014-8495-2. PMC4106675 24614953

[51] M. J. Jedrzejas , L. V. Mello , B. L. de Groot , and S. Li , “Mechanism of Hyaluronan Degradation by *Streptococcus pneumoniae* Hyaluronate Lyase: Structures of Complexes With the Substrate,” Journal of Biological Chemistry 277, no. 31 (2002): 28287–28297, 10.1074/jbc.M203844200. 11991948

[52] U. Eckhard , P. F. Huesgen , H. Brandstetter , and C. M. Overall , “Proteomic Protease Specificity Profiling of Clostridial Collagenases Reveals Their Intrinsic Nature as Dedicated Degraders of Collagen,” Journal of Proteomics 100 (2014): 102–114, 10.1016/j.jprot.2013.10.004. PMC3985423 24125730

[53] N. K. Karamanos , Z. Piperigkou , A. D. Theocharis , et al., “Proteoglycan Chemical Diversity Drives Multifunctional Cell Regulation and Therapeutics,” Chemical Reviews 118, no. 18 (2018): 9152–9232, 10.1021/acs.chemrev.8b00354. 30204432

[54] R. V. Iozzo and L. Schaefer , “Proteoglycan Form and Function: A Comprehensive Nomenclature of Proteoglycans,” Matrix Biology 42 (2015): 11–55, 10.1016/j.matbio.2015.02.003. PMC4859157 25701227

[55] L. Schaefer and R. M. Schaefer , “Proteoglycans: From Structural Compounds to Signaling Molecules,” Cell and Tissue Research 339, no. 1 (2010): 237–246, 10.1007/s00441-009-0821-y. 19513755

[56] I. Vlodavsky , N. Ilan , A. Naggi , and B. Casu , “Heparanase: Structure, Biological Functions and Inhibition by Heparin‐Derived Mimetics,” FEBS Journal 274, no. 10 (2007): 2554–2561, 10.1111/j.1742-4658.2007.05794.x. 17627539

[57] C. R. Parish , C. Freeman , and M. D. Hulett , “Heparanase: A Key Enzyme Involved in Cell Invasion,” Biochimica et Biophysica Acta 1471, no. 3 (2001): M99–M108, 10.1016/S0304-4165(00)00135-2. 11250066

[58] M. King , C. Convery , and E. Davies , “The Use of Hyaluronidase in Aesthetic Practice,” Journal of Clinical and Aesthetic Dermatology 11, no. 6 (2018): E61–E68. PMC6011868 29942426

[59] K. Goldie , W. Peeters , M. Alghoul , et al., “Global Consensus Guidelines for the Injection of Diluted and Hyperdiluted Calcium Hydroxylapatite for Skin Tightening,” Dermatologic Surgery 44, no. Suppl 1 (2018): S32–S41, 10.1097/DSS.0000000000001685. 30358631

[60] M. O. Christen and F. Vercesi , “Polycaprolactone: How a Well‐Known and Futuristic Polymer Has Become an Innovative Collagen Stimulator in Esthetics,” Clinical, Cosmetic and Investigational Dermatology 13 (2020): 31–48, 10.2147/CCID.S229054. PMC7065466 32161484

[61] R. Fitzgerald and D. Vleggaar , “Facial Volume Restoration of the Aging Face With Poly‐L‐Lactic Acid,” Dermatologic Therapy 24, no. 1 (2011): 2–27, 10.1111/j.1529-8019.2010.01375.x. 21276155

[62] C. F. Schierle and L. A. Casas , “Nonsurgical Rejuvenation of the Aging Face With Injectable Poly‐L‐Lactic Acid for Restoration of Soft Tissue Volume,” Aesthetic Surgery Journal 31, no. 1 (2011): 95–109, 10.1177/1090820X10391213. 21239677

[63] D. S. Orentreich and N. Orentreich , “Subcutaneous Incisionless Surgery for the Correction of Depressed Scars and Wrinkles,” Dermatologic Surgery 21, no. 6 (1995): 543–549, 10.1111/j.1524-4725.1995.tb00259.x. 7773602

[64] M. C. Aust , D. Fernandes , P. Kolokythas , H. M. Kaplan , and P. M. Vogt , “Percutaneous Collagen Induction Therapy: An Alternative Treatment for Scars, Wrinkles, and Skin Laxity,” Plastic and Reconstructive Surgery 121, no. 4 (2008): 1421–1429, 10.1097/01.prs.0000304612.72899.02. 18349665

[65] B. Berman and F. Flores , “The Treatment of Hypertrophic Scars and Keloids,” European Journal of Dermatology 7, no. 8 (1997): 591–595. 9889433

[66] B. S. Atiyeh , “Nonsurgical Management of Hypertrophic Scars: Evidence‐Based Therapies, Standard Practices and Emerging Methods,” Aesthetic Plastic Surgery 31, no. 5 (2007): 468–492, 10.1007/s00266-006-0253-y. 17576505

[67] M. P. Baker , H. M. Reynolds , B. Lumicisi , and C. J. Bryson , “Immunogenicity of Protein Therapeutics: The Key Causes, Consequences and Challenges,” Self/Nonself 1, no. 4 (2010): 1–17, 10.4161/self.1.4.13904. PMC3062386 21487506

[68] S. Hermeling , H. Schellekens , D. J. A. Crommelin , and W. Jiskoot , “Micelle‐Associated Protein in Epoetin Formulations: A Risk Factor for Immunogenicity?,” Pharmaceutical Research 20, no. 12 (2003): 1903–1907, 10.1023/B:PHAM.0000008034.61317.02. 14725351

[69] F. H. Arnold , “Directed Evolution: Bringing New Chemistry to Life,” Angewandte Chemie (International Ed. in English) 57, no. 16 (2018): 4143–4148, 10.1002/anie.201708408. PMC5901037 29064156

[70] F. S. Collins and H. Varmus , “A New Initiative on Precision Medicine,” New England Journal of Medicine 372, no. 9 (2015): 793–795, 10.1056/NEJMp1500523. PMC5101938 25635347

[71] E. A. Ashley , “The Precision Medicine Initiative: A New National Effort,” Journal of the American Medical Association 313, no. 21 (2015): 2119–2120, 10.1001/jama.2015.3595. 25928209

[72] D. G. Castelanich , L. A. Parra Hernández , and M. Chacín , “Successfully Nonsurgical Epidermoid Cyst Management With Recombinant Hydrolytic Enzymes: A Case Report,” Clinical, Cosmetic and Investigational Dermatology 17 (2024): 2501–2508, 10.2147/CCID.S469175. PMC11321352 39139846

